# AMMI-Bayesian perspective in the selection of pre-cultivars of carioca beans in *Agreste-Sertão* of Pernambuco, Brazil

**DOI:** 10.1038/s41598-023-31768-5

**Published:** 2023-03-22

**Authors:** Gérsia Gonçalves de Melo, Luciano Antonio de Oliveira, Carlos Pereira da Silva, Alessandra Querino da Silva, Maxwel Rodrigues Nascimento, Ranoel José de Sousa Gonçalves, Paulo Ricardo dos Santos, Antônio Félix da Costa, Damião Ranieri Queiroz, José Wilson da Silva

**Affiliations:** 1 Universidade Federal Rural de Pernambuco, Recife, PE Brazil; 2 Universidade Federal da Grande Dourados, Dourados, MS Brazil; 3 Universidade Federal de Lavras, Lavras, MG Brazil; 4 Universidade Estadual Do Norte Fluminense Darcy Ribeiro, Campos dos Goytacazes, RJ Brazil; 5 Universidade Federal de Campina Grande, Sumé, PB Brazil; 6 Instituto Federal do Amapá, Porto Grande, AP Brazil; 7 Instituto Agronômico de Pernambuco, Recife, PE Brazil

**Keywords:** Genetics, Agricultural genetics

## Abstract

The productivity of beans is greatly influenced by the different edaphoclimatic conditions in the Agreste-Sertão region, requiring the identification of adapted and stable genotypes to minimize the effects of the interaction between genotypes per environments (GxE). The objective of this work was to analyze the adaptability and stability of carioca bean pre-cultivars in three municipalities in the Agreste-Sertão of Pernambuco using the AMMI model in its Bayesian version BAMMI and compare the results with the frequentist approach. According to the results, the BAMMI analysis showed better predictive capacity, as well as better performance in the study of adaptability and stability. The cultivar BRS Notável stood out in terms of main effect and stability. Adaptability of genotypes to specific locations was also observed, enabling the use of the positive effect of the GxE interaction, which was more evident with the BAMMI model. From this work, the flexibility of BAMMI model to deal with data resulting from multi-environmental experiments can be seen, overcoming limitations of the standard analysis of the AMMI model.

## Introduction

The common bean (*Phaseolus vulgaris* L.) is a legume highly valued and cultivated worldwide, mainly due to its high nutritional quality, being an important source of protein in human food^1^. In Brazil, this culture has great socioeconomic importance and stands out in subsistence agriculture, being cultivated in different environments and at different technological levels of production^2^.

Brazil occupies the first and third place in the world ranking of consumption and production of beans, respectively, with annual production of 2.89 million tons. In most states of the country, preference is observed for the carioca commercial group, which represents about 60% of national consumption^3^.

In the *Agreste-Sertão* of Pernambuco, bean cultivation is carried out in several municipalities, which cover different edaphoclimatic conditions and, consequently, influence directly on productivity^4^. In this sense, the interaction between genotypes x environments (GxE) is an important challenge for breeders in the evaluation of pre-cultivars since the environment can mask the true potential of the genotype^5^.

Minimization of the effects of the GxE interaction can be achieved by identifying the most stable genotypes (with wide recommendation) and/or by identifying the adaptability of genotypes to specific environments. The evaluation of genotypic stability and adaptability can be conducted using different statistical methodologies^6–8^.

The AMMI (Additive Main effects and Multiplicative Interaction analysis) model is one of the most popular frequentist methods for analyzing genotype responses in various environments^8–10^. This model offers several advantages, among which its good predictive capacity and the possibility of graphically describing the effect of the interaction in biplots stand out, making data interpretation simpler^11–13^.

Despite the advantages offered, the classic AMMI analysis has limitations that restrict or make its use unfeasible, such as the impossibility of working with unbalanced and/or heteroscedastic data sets, the requirement to work with these effects as fixed, e a dificuldade em construir estatísticas inferenciais para os parâmetros bilineares da interação GxE^9^.

It is worth mentioning that there are methods that circumvent the difficulties related to the data structure, using decomposition by singular value, such as imputation of missing values, corrections in the averages of the little houses and weighting in relation to the environments^14,15^. However, some of these procedures are susceptible to criticism and can lead to loss of information^12,16^.

To circumvent such problems, an alternative that has proved to be relevant is the application of the Bayesian approach to the AMMI model (Bayesian-AMMI or BAMMI). The BAMMI model offers flexibility in analyzing experimental data under different conditions, demonstrating viability for use in balanced data^10,17^, unbalanced^9^, heteroscedastic^16^, with a posteriori fixed effect for all parameters^18^ and with random effect for genotypes and/or singular values^11,19^.

The Bayesian approach allows the incorporation of prior information, when it is available, enabling greater efficiency in the analysis^16,20–22^. However, there are still few studies exploring the predictive power of BAMMI in relation to frequentist AMMI.

Romão et al.^8^ investigated the predictive power between AMMI via EM algorithm (EM-AMMI), Bayesian AMMI with homogeneity (BAMMI) and heterogeneity of variances (BAMMI-H) and the Analytical Factorial (FA) model, but using simulated data. Thus, this work is a pioneer in the investigation of the predictive capacity of BAMMI in relation to the frequentist AMMI using real data. Furthermore, it innovates by employing the ammiBayes statistical package, recently developed by Oliveira et al.^23^, for the inference process with the BAMMI model.

Therefore, this work aimed to analyze the adaptability and stability of carioca bean pre-cultivars in the *Agreste-Sertão* of Pernambuco using the AMMI model (Additive Main effects and Multiplicative Interaction analysis) in its Bayesian version BAMMI (Bayesian AMMI) and comparing the results with the frequentist approach.

## Material and methods

### Sample data and experimental conditions

**Figure 1:**
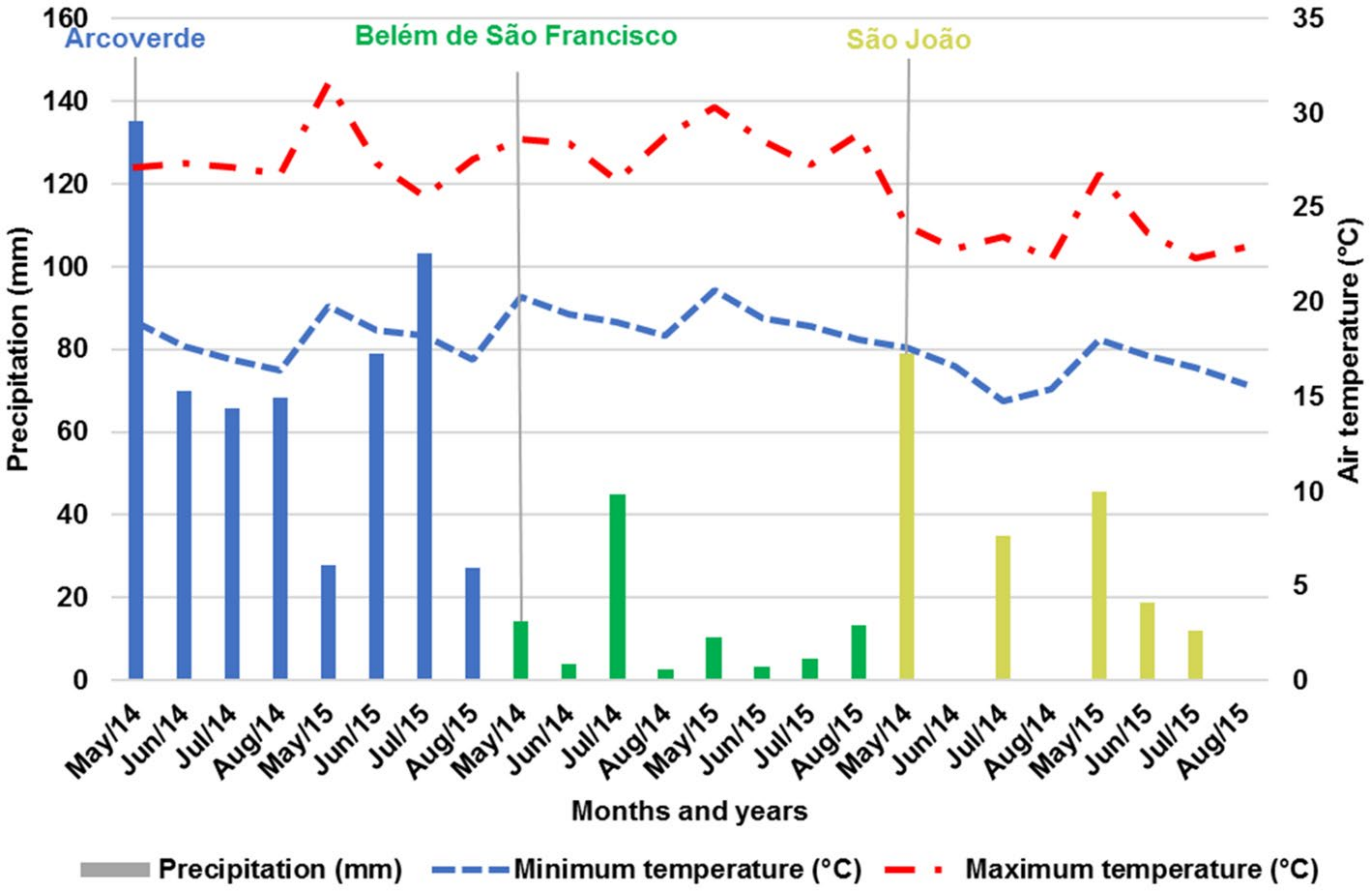
Graph of the climatic conditions of the municipalities of Pernambuco, where the carioca bean genotypes were evaluated, in the years 2014 and 2015.

Grain productivity data (kg ha^-1^) are from competition trials of common bean pre-cultivars of the carioca type, performed in three municipalities in the *Agreste-Sertão* of Pernambuco (Table 1), in the period from May to August of the years 2014 and 2015, conducted by the Pernambuco Agronomic Institute (IPA) and the Federal Rural University of Pernambuco (UFRPE). These municipalities have different climatic conditions, incurring a greater representation of the region (Fig. 1).

**Table 1:** Characteristics of the municipalities in Pernambuco, where the carioca bean genotypes were evaluated, in the years 2014 and 2015.

Municipalities	Topography	Climate	Alt	Soil type	Year 2014		Year 2015	
					Prec	Temp	Prec	Temp
Arcoverde	Wavy	BSh	689	No. Regolith	192.5	24.3	203	25.2
Belém de S.F	Wavy S	BSh	339	Flat Ground	400#	25.6	450#	26.2
São João	Wavy	As	687	No. Regolith	318.8	21.2	333.3	22

*S.F.* São Francisco, *S.* Slightly, *As* Tropical, *BSh* Semi-arid^24^, *Alt.* Altitude (m), *N.* Neosol, *Prec.* Average rainfall in the growing season (mm), # Irrigated system, *Temp.* Temperature (°C).

The genotypes used consisted of 10 lineages and four cultivars, from the National Rice and Beans Research Center (CNPAF) from the Brazilian Agricultural Research Corporation (Embrapa Rice and Beans) (Table 2). The cultivars were used as witnesses in the experimental tests since they are recommended for cultivation in the state of Pernambuco.

**Table 2:** Carioca bean genotypes evaluated in competition trials in the years 2014 and 2015, treatment identification number (IG).

IG	Genotype	Type	IG	Genotype	Type
G1	IPR 139	Cultivar	G8	CNFC 15504	Lineage
G2	CNFC 15458	Lineage	G9	CNFC 15507	Lineage
G3	CNFC 15460	Lineage	G10	CNFC 15513	Lineage
G4	CNFC 15462	Lineage	G11	CNFC 15534	Lineage
G5	CNFC 15475	Lineage	G12	BRS Estilo	Cultivar
G6	CNFC 15480	Lineage	G13	BRS Notável	Cultivar
G7	CNFC 15497	Lineage	G14	BRS Pérola	Cultivar

The experimental design was in randomized blocks, with three replications. The experimental plots consisted of four rows spaced 0.5 × 0.1 m between and inside, respectively, and the useful area consisted of the two central rows.

Soil preparation was done in a conventional way, with chemical fertilization with 40 kg ha^**-1**^ of N, 60 kg ha^-1^ of P_2_O_5_ and 30 kg ha^-1^ of K_2_O before the implementation of the tests. Irrigation was performed when necessary for cultivation in Belém de São Francisco, using a conventional sprinkler system, because in the others the rainfed system predominated. Weed plants control was done by hand weeding and for pest control, application of Metamidophos Fersol (600 at a dose of 0.5 L ha^-1^) was promoted. The measurement of the grain productivity variable grain productivity experiment, and the harvest was carried out 90 days after planting, in the R9 phase.

### Statistical analysis

The AMMI model in matrix notation, as presented in Oliveira et al.^10^, is given by:

1 $${\mathbf{y}} = {\mathbf{X}}_{1} {\varvec{\beta}} + {\mathbf{Z}}{\varvec{g}} + \mathop \sum \limits_{k = 1}^{t} \lambda_{k} diag({\mathbf{Z}}{\varvec{\alpha}}_{k} ){\mathbf{X}}_{2} {\varvec{\gamma}}_{k} + {\varvec{\varepsilon}}$$

being ${\mathbf{y}}_{n \times 1}$ the vector composed of phenotypic response, where $l$, *r* and *c* denote, respectively, the number of repetitions, the number of genotypes and the number of environments. The vectors ${\varvec{\beta}}_{cl\, \times 1}$ and ${\varvec{g}}_{r \times 1}$ contain the effects parameters of hierarchical blocks within environments and main effects of genotypes, respectively.

The terms ${\lambda }_{k}$, ${\boldsymbol{\alpha }}_{{\varvec{k}}}$ and ${{\varvec{\gamma}}}_{{\varvec{k}}}$ are the multiplicative or bilinear components of model (1) and denote, respectively, the singular value and the singular vectors associated with the *k*-th principal component, with $k = \,1,\,...,\,t$ being $t = \min \,(r,\,c)$ the rank of the "GxE" interaction matrix. Bilinear components are also subject to order restrictions ($\lambda_{1} > \lambda_{2} > \cdots > \lambda_{t}$) and orthonormalization $\left({\boldsymbol{\alpha }}_{k}^{\top }{\boldsymbol{\alpha }}_{k}={{\varvec{\gamma}}}_{k}^{\top }{{\varvec{\gamma}}}_{k}=1 e{\boldsymbol{ }\boldsymbol{\alpha }}_{k}^{\top }{\boldsymbol{\alpha }}_{{k}^{^{\prime}}}={{\varvec{\gamma}}}_{k}^{\top }{{\varvec{\gamma}}}_{{k}^{^{\prime}}}=0;k\ne {k}^{^{\prime}}\right)$.

The matrices ${\mathbf{X}}_{1}$, ${\mathbf{X}}_{2}$ and ${\mathbf{Z}}$ are design matrices associated with ${\varvec{\beta}}$, ${{\varvec{\upgamma}}}_{k}$ and ${\varvec{g}}$, respectively. The term ${\varvec{\varepsilon}}$ is the error vector, with $\user2{\varepsilon \sim }N_{n} ({\mathbf{0}},\,\sigma_{e}^{2} {\mathbf{I}}_{n} )$, where ${\mathbf{0}}$ represents the null vector and ${\mathbf{I}}_{n}$ the identity matrix of order n. The conditional distribution of ${\mathbf{y}}$ is multivariate normal, that is, ${\varvec{y}}|{\varvec{\alpha}},{\varvec{\gamma}},\lambda , {\varvec{g}},{\varvec{\beta}},\sigma_{e}^{2} \sim N_{n} \left( {{{\varvec{\upmu}}},{\mathbf{I}}_{n} \sigma_{e}^{2} } \right)$ with ${{\varvec{\upmu}}} = {\mathbf{X}}_{1} {\varvec{\beta}} + {\mathbf{Z}}{\varvec{g}} + \mathop \sum \limits_{k = 1}^{t} \lambda_{k} diag({\mathbf{Z}}{\varvec{\alpha}}_{k} ){\mathbf{X}}_{2} {\varvec{\gamma}}_{k}$, being $\sigma_{e}^{2}$ the residual variance.

### A priori* distributions and posterior conditional distributions*

The a priori distributions used for the parameters of model (1) are the same used by Oliveira et al.^10^:

$${{\varvec{\upbeta}}}{|}\,{{\varvec{\upmu}}}_{{{\varvec{\upbeta}}}} ,\,\sigma_{{{\varvec{\upbeta}}}}^{2} \sim \,N\left( {{{\varvec{\upmu}}}_{\beta } ,\,\sigma_{{{\varvec{\upbeta}}}}^{2} } \right)\,$$;

$${\mathbf{g}}|{{\varvec{\upmu}}}_{{\mathbf{g}}} ,\sigma_{{\mathbf{g}}}^{2} \sim N({{\varvec{\upmu}}}_{{\mathbf{g}}} ,{\mathbf{I}}\sigma_{{\mathbf{g}}}^{2} )$$;

$$\lambda_{k} |\mu_{{\lambda_{k} }} ,\sigma_{{\lambda_{k} }}^{2} \sim N^{ + } (\mu_{{\lambda_{k} }} ,\sigma_{{\lambda_{k} }}^{2} )$$;

${\varvec{\alpha}}_{k}$ ~ uniform spherical distribution;

${\varvec{\gamma}}_{k}$ ~ uniform spherical distribution;

$$\sigma_{e}^{2} \sim {1 \mathord{\left/ {\vphantom {1 {\sigma_{e}^{2} }}} \right. \kern-0pt} {\sigma_{e}^{2} }} .$$

For Gaussian distributions, a priori information can be incorporated by assigning values for average and variance. In order to incorporate minimum information, it was considered ${{\varvec{\upmu}}}_{{{\varvec{\upbeta}}}} = {\mathbf{0}}$, $\mu_{{\lambda_{k} }} = 0$, $\sigma_{\beta }^{2} = 10^{8}$ and $\sigma_{{\lambda_{k} }}^{2} = 10^{8}$. To the singular vectors ${{\varvec{\upalpha}}}_{k}$ and ${{\varvec{\upgamma}}}_{k}$ uniform spherical a priori densities were assigned in the corrected subspace, which are uninformative^25^ and for the experimental variance an a priori of Jeffrey was assigned $\sigma_{e}^{2} = {1 \mathord{\left/ {\vphantom {1 {\sigma_{e}^{2} }}} \right. \kern-0pt} {\sigma_{e}^{2} }}$. For the genotype effect, ${{\varvec{\upmu}}}_{{\mathbf{g}}} = {\mathbf{0}}$ and $\sigma_{g}^{2} \sim {1 \mathord{\left/ {\vphantom {1 {\sigma_{g}^{2} }}} \right. \kern-0pt} {\sigma_{g}^{2} }}$ were considered, obtaining a posteriori random effect for genotypes. This model is the same used by Oliveira et al.^10^, being referred to by BAMMI (Bayesian-AMMI).

Complete conditional distributions are described in detail in Oliveira et al.^10^ or Silva et al.^19^ and are the following:

$${{\varvec{\upbeta}}}| \ldots \sim N\left[ {\left( {{\mathbf{X}}_{1}^{{{\top}}} {\mathbf{X}}_{1} + {\mathbf{I}}\frac{1}{{\sigma_{{{\varvec{\upbeta}}}}^{2} }}} \right)^{ - 1} {\mathbf{X}}_{1}^{{{\top}}} {\mathbf{M}}_{{{\varvec{\upbeta}}}} ,\;\left( {{\mathbf{X}}_{1}^{{{\top}}} {\mathbf{X}}_{1} + {\mathbf{I}}\frac{1}{{\sigma_{{{\varvec{\upbeta}}}}^{2} }}} \right)^{ - 1} } \right]$$, where $$\sigma_{{{\varvec{\upbeta}}}}^{2} = 10^{8}$$ and.

$${\mathbf{M}}_{{{\varvec{\upbeta}}}} = {\mathbf{y}} - {\mathbf{Zg}} - \sum\nolimits_{k = 1}^{t} {\lambda_{k} diag({\mathbf{Z\alpha }}_{k} ){\mathbf{X}}_{2} {{\varvec{\upgamma}}}_{k} }$$.

$${\mathbf{g}}|...\sim N\left[ {\left( {{\mathbf{Z}}^{{{\top}}} {\mathbf{Z}} + {\mathbf{I}}\frac{1}{{\sigma_{{\mathbf{g}}}^{2} }}} \right)^{ - 1} {\mathbf{Z}}^{{{\top}}} {\mathbf{M}}_{{\mathbf{g}}} \,,\,\,\,\,\left( {{\mathbf{Z}}^{{{\top}}} {\mathbf{Z}} + {\mathbf{I}}\frac{1}{{\sigma_{{\mathbf{g}}}^{2} }}} \right)^{ - 1} } \right]$$$${\mathbf{M}}_{{\mathbf{g}}} = {\mathbf{y}} - {\mathbf{X}}_{1} {{\varvec{\upbeta}}} - \sum\nolimits_{k = 1}^{t} {\lambda_{k} diag({\mathbf{Z\alpha }}_{k} ){\mathbf{X}}_{2} {{\varvec{\upgamma}}}_{k} }$$ where:

$${\sigma }_{{\varvec{g}}}^{2}|...\sim \mathit{inv}-{\chi }^{2}\left({n}_{g},{{\varvec{g}}}^{\mathrm{\top }}{\varvec{g}}\right)$$

$${ }\lambda_{k} | \ldots \sim N^{ + } \left[ {\left( {{{\varvec{\Lambda}}}_{k}^{{{\top}}} {{\varvec{\Lambda}}}_{k} + \frac{{\sigma_{e}^{2} }}{{\sigma_{{\lambda_{k} }}^{2} }}} \right)^{ - 1} {{\varvec{\Lambda}}}_{k}^{{{\top}}} {\mathbf{\rm M}}_{k^{\prime}}^{{{\top}}} , \,\,\left( {{{\varvec{\Lambda}}}_{k}^{{{\top}}} {{\varvec{\Lambda}}}_{k} } \right)^{ - 1} \sigma_{e}^{2} } \right]$$ being $${{\varvec{\Lambda}}}_{k} = diag({\mathbf{Z\alpha }}_{k} ){\mathbf{X}}_{2} {{\varvec{\upgamma}}}_{k}$$;$${\mathbf{\rm M}}_{k^{\prime}}^{{}} = {\mathbf{y}} - {\mathbf{Zg}} - {\mathbf{X}}_{1} {{\varvec{\upbeta}}} - \mathop \sum \limits_{k^{\prime} \ne k}^{t} \lambda_{k^{\prime}} diag({\mathbf{Z\alpha }}_{k^{\prime}} ){\mathbf{X}}_{2} {{\varvec{\upgamma}}}_{k^{\prime}}$$ and $$\lambda_{1} \ge \ldots \ge \lambda_{t} \ge 0.$$

$$p({{\varvec{\upalpha}}}_{k} | \cdots ) \propto exp\left\{ {\frac{{\lambda_{k} }}{{\sigma_{e}^{2} }}\left[ {{{\varvec{\upalpha}}}_{k}^{{{\top}}} {{\varvec{\Lambda}}}_{{{{\varvec{\upalpha}}}_{k} }}^{{{\top}}} \left( {{\mathbf{y}} - {\mathbf{X}}_{1} {{\varvec{\upbeta}}}} \right)} \right]} \right\}$$

being $${{\varvec{\Lambda}}}_{{{{\varvec{\upalpha}}}_{k} }} = diag\left( {{\mathbf{X}}_{2} {{\varvec{\upgamma}}}_{k} } \right){\mathbf{Z}}$$, and

$$p({{\varvec{\upgamma}}}_{k} | \cdots ) \propto exp\left\{ {\frac{{\lambda_{k} }}{{\sigma_{e}^{2} }}\left[ {{{\varvec{\upgamma}}}_{k}^{{{\top}}} {{\varvec{\Lambda}}}_{{{{\varvec{\upgamma}}}_{k} }}^{{{\top}}} \left( {{\mathbf{y}} - {\mathbf{X}}_{1} {{\varvec{\upbeta}}}} \right)} \right]} \right\}$$

being $${{\varvec{\Lambda}}}_{{{{\varvec{\upgamma}}}_{k} }} = diag\left( {{\mathbf{Z\alpha }}_{k} } \right){\mathbf{X}}_{2}$$.

The a posteriori densities for the singular vectors are proportional to spherical distributions of the von Mises-Fisher type (vMF). Due to the orthogonality constraints of the singular vectors the a posteriori distribution for these parameters is non-trivial and sampling must be performed by auxiliary variables in the corrected subspace. Details on this process are presented in Viele and Srinivasan^25^, Liu^26^ and Oliveira et al.^10^.

Finally, the complete a posteriori conditional distribution for the residual variance is inverse scaling chi-square:

$$\sigma_{{\varvec{e}}}^{2} |...\sim inv - \chi^{2} \left( {n,\frac{{\left( {{\varvec{y}} - {\varvec{\mu}}} \right)^{{{\top}}} \left( {{\varvec{y}} - {\varvec{\mu}}} \right)}}{n}} \right)$$

### MCMC sampling, model selection and comparison

The Markov chains, as well as the entire inference process with the BAMMI model, were obtained using the ammiBayes package^23^. The sampling of parameters was conducted using a Gibbs sampler and a description of the iterative algorithm can be found in Oliveira et al.^10^. The convergence of the produced chains was monitored by the criteria of Raftery and Lewis^27^ and Heidelberger and Welch^28^, using the library Bayesian Output Analysis (BOA)^29^.

Model selection was performed using BIC^30^, AIC^31^ and AICM^32^ information criteria. The estimates for the univariate parameters of the model were obtained by a posteriori averages of the MCMC chains of regions of maximum a posteriori density (HPD), built using the BOA package^29^. Bivariate regions at 95% credibility, for genotypic and environmental scores in the biplot representation, were implemented using the method of Hu and Yang^33^.

The classic AMMI (frequentist approach) was adjusted for comparison purposes. minimum squares method for the estimation of the effects and the multiplicative terms of the interaction, estimated from the singular value decomposition (DVS)^34^. Model selection was performed using Cornelius' Fr test^35^.

Random imbalances were performed in the data, with levels of 10% and 20% loss of genotypes and environments, to verify the efficiency of the methods, and later the analyzes were performed. As the analyzes were conducted in two imbalance scenarios the EM (expectation–maximization) algorithm was used to impute the missing values in the AMMI-Classic approach^36^.

An assessment of predictive ability was performed for the BAMMI and the AMMI frequentist models using cross-validation procedures. To compare the two models, the correlation between predicted and observed values (Cor), Spearman's Correlation (CorS) and PRESS (Prediction Error Sum Square) was used. Statistical analyzes were performed considering each location-year combination as an environment and were performed using the R statistical software^37^.

## Results

### AMMI-classic analysis

The joint analysis showed a significant effect of the interaction, indicating non-additivity of the main effects, justifying the application of the AMMI analysis (Table 3).

**Table 3:** Analysis of the joint variance of competition trials of carioca bean pre-cultivars, evaluated in Agreste-Sertão of Pernambuco, from 2014 to 2015.

Sources of variation	GL	Sum of squares	Medium squares	*F* value
Blocks / Environments	12	0.98	0.08	1.39
Environments	5	75.48	15.10	30.15***
Genotypes	13	14.54	1.12	2.23*
G x A	65	32.54	0.50	8.51***
Residue	156	9.17	0.06	
Total	251	132.72		
Average (kg ha^-1^)			2203.62	
CV (%)			11	

* and *** significant at 5% and 0.1% probability by the F test, respectively.

According to Cornelius' Fr test, the model that best described the data set was AMMI-4, that is, the model that presents four main components retained to explain the effect of the GxE interaction. In this model, 98% of the variation of the GxE interaction is explained (Table 4).

**Table 4:** Cornelius' Fr test results from AMMI analysis for competition trials of carioca bean pre-cultivars, evaluated in Agreste-Sertão of Pernambuco, from 2014 to 2015.

Models	GL	Sum of squares	Medium squares	%	% Accumulated	F value
AMMI1	48	12.99	0.41	39.92	39.92	6.92***
AMMI2	33	7.97	0.35	24.49	64.41	5.97***
AMMI3	20	6.61	0.25	20.32	84.72	4.22***
AMMI4	9	4.31	0.07	13.26	97.99	1.24
AMMI5	0	0.65	0	2.01	100	1

*** significant at 0.1% probability by the F test.

**Figure 2:**
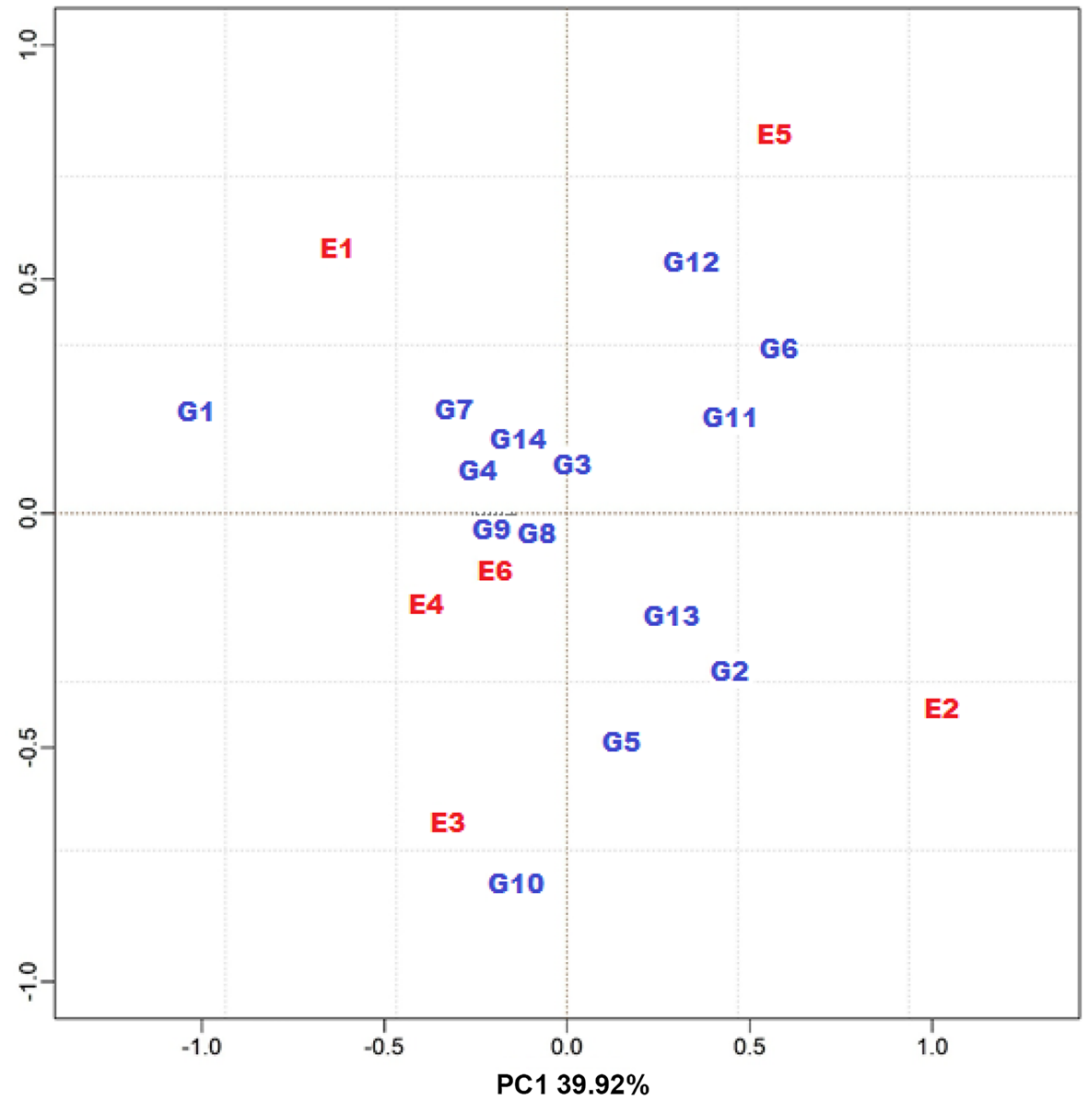
AMMI2 model biplot with average genotypic and environmental scores for data from competition trials with carioca beans in the Agreste-Sertão region of Pernambuco.

Although the AMMI-4 model was the one that best fit the data, in the analysis of adaptability and stability, the most common is the use of the AMMI-2 biplot, since in the first axes are the highest percentages referring to the interaction pattern, with less influence of noise, even when they explain a lower percentage of the sum of squares^38^. The AMMI-2 biplot representation of the genotypic and environmental scores is shown in Fig. 2.

The lineages CNFC 15460 (G3), CNFC 15462 (G4), CNFC 15504 (G8), CNFC 15507 (G9) and the cultivar BRS Pérola (G14), were closer to the origin and, therefore, are the ones that contributed the least to the interaction effect, demonstrating wide adaptability, as well as the environments Arcoverde-2015 (E4) and São João-2015 (E6). On the other hand, IPR 39 (G1), CNFC 15513 (G10) and BRS Estilo (G12) were further away from the center of the biplot and that is why they are considered the most unstable (Fig. 2). The smallest contribution visualized in the interaction axis points to greater stability and indicates that the average productivity of these genotypes is little variable, depending on locations and years^39^.

The behavior of some genotypes within the same location was incongruous between the two years of evaluation. This can be visualized by taking as an example the Belém de São Francisco municipality, in which specific adaptability of the genotypes CNFC 15458 (G2) and BRS Notável (G13) was suggested in the year 2014 (E2), whereas in 2015 (E5) this was observed in relation to another material, the BRS Estilo (G12) (Fig. 2).

In relation to the Arcoverde municipality, the genotype CNFC 15497 (G7) showed specific adaptability, in the year 2014 (E1), while in 2015 (E4) there was no significant contribution from the environment to the interaction (Fig. 2).

The genotypes CNFC 15475 (G5) and CNFC 15513 (G10) showed specific adaptability to the municipality São João in 2014 (E3), which in turn did not contribute significantly to the interaction in 2015 (E6). In addition to the stable genotypes, CNFC15513 (G10) would be the most interesting for this specific location in terms of recommendation (Fig. 2).

### AMMI-bayesian analysis

For all MCMC chains sampled, good convergence properties were observed from the criteria used, since all model parameters had a dependency factor lower than 5^27^ and passed the stationarity test^28^.

**Figure 3:**
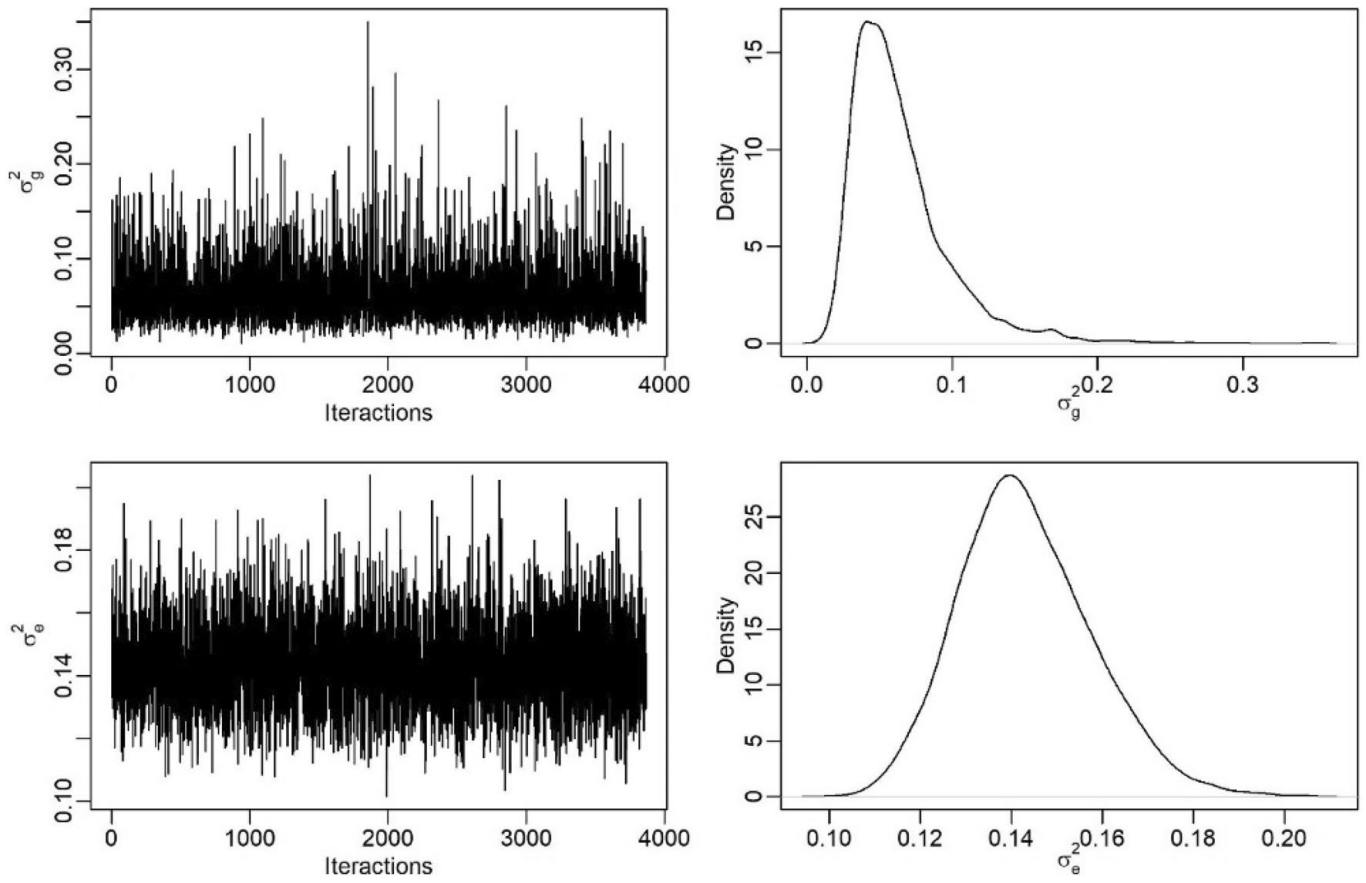
Trace plots and MCMC chain densities for genotypic and residual variance of data from competition trials with carioca beans in the Agreste-Sertão region of Pernambuco.

In the graphs of traces and densities of the MCMC chains, it was observed that the distributions were stationary, corroborating the results obtained by the applied convergence tests. For examples, Fig. 3 shows the plot of traces for the genotypic and residual variance of the BAMMI-1 model, which was the model that best fitted the data in the selection stage.

In Table 5, point and interval estimates related to singular values are presented for BAMMI models as a function of the number of bilinear components retained. Based on the complete BAMMI-5 model, it can be stated that the first axis explains 42.8% of all the variation in the interaction.

**Table 5:** A posteriori average, a posteriori standard deviation, and HPD interval (95% credibility) for the singular value referring to the possible models for approaches.

Model	Par	Average	Sd	LL	UL
BAMMI-1	${\lambda }_{1}$	1.87	0.23	1.41	2.31
BAMMI-2	${\lambda }_{1}$	1.93	0.20	1.54	2.30
BAMMI-2	${\lambda }_{2}$	1.49	0.19	1.10	1.84
BAMMI-3	${\lambda }_{1}$	1.97	0.17	1.65	2.30
BAMMI-3	${\lambda }_{2}$	1.49	0.17	1.16	1.82
BAMMI-3	${\lambda }_{3}$	1.29	0.15	1	1.60
BAMMI-4	${\lambda }_{1}$	2	0.15	1.70	2.28
BAMMI-4	${\lambda }_{2}$	1.53	0.14	1.24	1.80
BAMMI-4	${\lambda }_{3}$	1.33	0.13	1.08	1.58
BAMMI-4	${\lambda }_{4}$	1.08	0.13	0.83	1.35
BAMMI-5	${\lambda }_{1}$	2	0.14	1.71	2.28
BAMMI-5	${\lambda }_{2}$	1.52	0.14	1.24	1.80
BAMMI-5	${\lambda }_{3}$	1.33	0.13	1.07	1.58
BAMMI-5	${\lambda }_{4}$	1.07	0.14	0.80	1.34
BAMMI-5	${\lambda }_{5}$	0.26	0.15	0	0.53

*Sd* Standard deviation, *LL* Lower limit, *UL* Upper limit.

**Figure 4:**
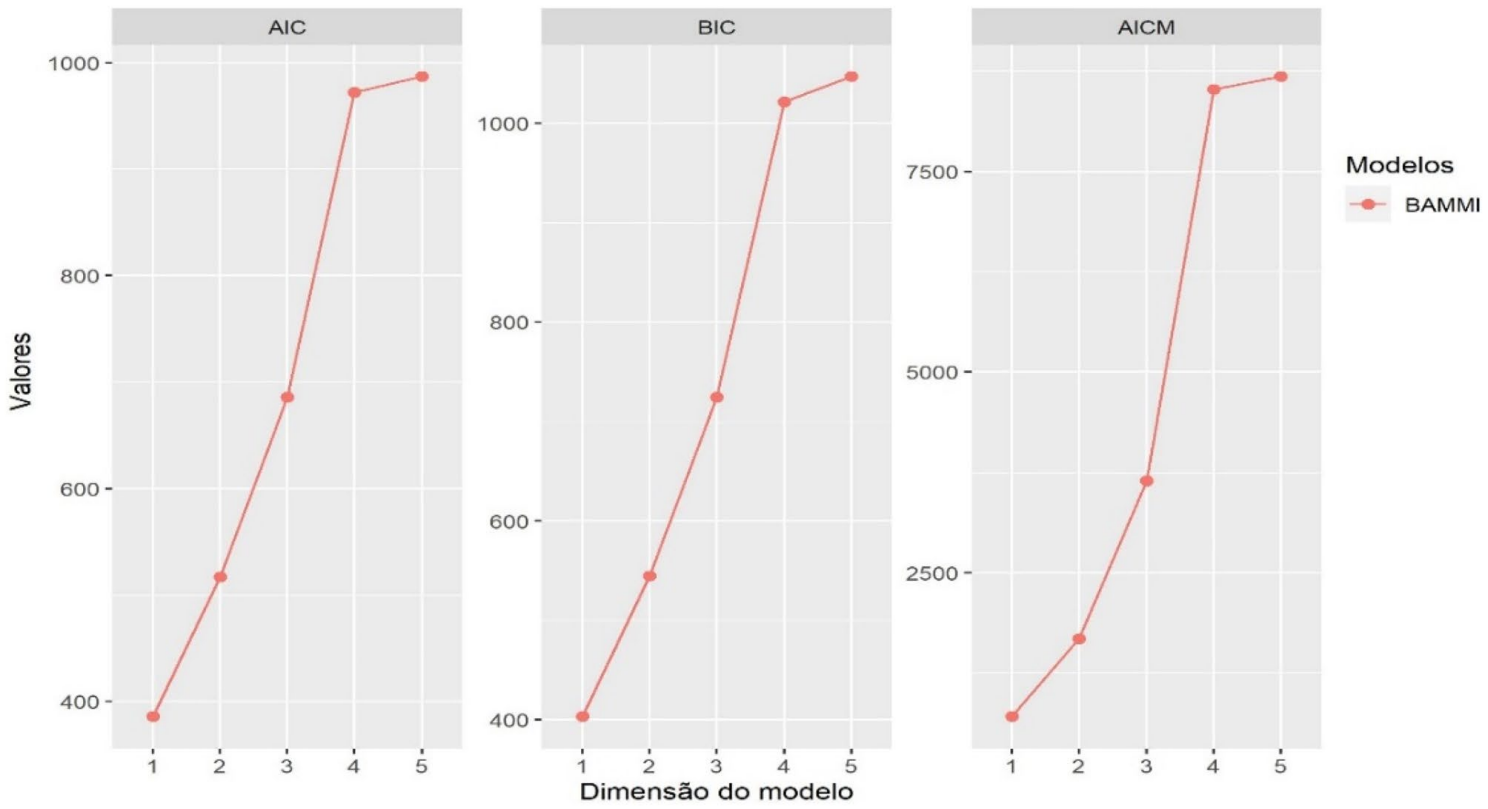
Graphics of the AIC, BIC and AICM information criteria for selecting the BAMMI model for data from competition trials with carioca beans in the Agreste-Sertão region of Pernambuco.

Figure 4 shows the results of the information criteria applied in the model selection stage. For all the criteria used, the model that best described the dataset was BAMMI-1, that is, the one that presented the lowest value of AIC, BIC and AICM. The choice based on these criteria focuses on more parsimonious and robust models according to the different a priori distributions^12^.

Table 6 presents point and interval estimates for genotype effects referring to BAMMI, as well as the minimum squares estimates obtained by the frequentist AMMI adjustment for comparison purposes. As can be seen, in general, the predictions of the BAMMI model are slightly smaller than the respective minimum squares solutions. The genotypes that stood out the most regarding the main effect were CNFC 15480 (G6), BRS Estilo (G12) and BRS Notável (G13).

**Table 6:** Genotype main effect, fixed effect estimates, a posteriori average and standard deviation estimates and HPD interval (95% credibility) (BAMMI-1).

Par	Fixed	Average	Sd	LL	UL
G1	0.02	0.02	0.09	− 0.15	0.20
G2	0.15	0.14	0.09	− 0.03	0.32
G3	0.06	0.05	0.09	− 0.11	0.24
G4	0.01	0.01	0.09	− 0.17	0.18
G5	− 0.17	− 0.16	0.09	− 0.34	− 0
G6	0.38	0.36	0.09	0.20	0.54
G7	− 0.38	− 0.36	0.09	− 0.54	− 0.19
G8	− 0.08	− 0.08	0.09	− 0.25	0.10
G9	− 0.30	− 0.28	0.09	− 0.46	− 0.11
G10	− 0.33	− 0.30	0.09	− 0.47	− 0.13
G11	− 0.08	− 0.07	0.09	− 0.24	0.10
G12	0.43	0.41	0.09	0.22	0.58
G13	0.25	0.24	0.09	0.07	0.42
G14	0.05	0.05	0.09	− 0.12	0.23

*Sd* Standard deviation, *LL* Lower limit, *UL* Upper limit.

Overlaps between the HPD intervals suggest similar effects, however the lineage CNFC 15480 (G6) and the cultivars BRS Estilo (G12) and BRS Notável (G13) did not include negative values in their credibility regions and, therefore, are the most interesting in terms of the main effect (Table 6). Positive HPD values indicate that these genotypes contributed the most to the population average and consequently are the ones with the highest productivity.

**Figure 5:**
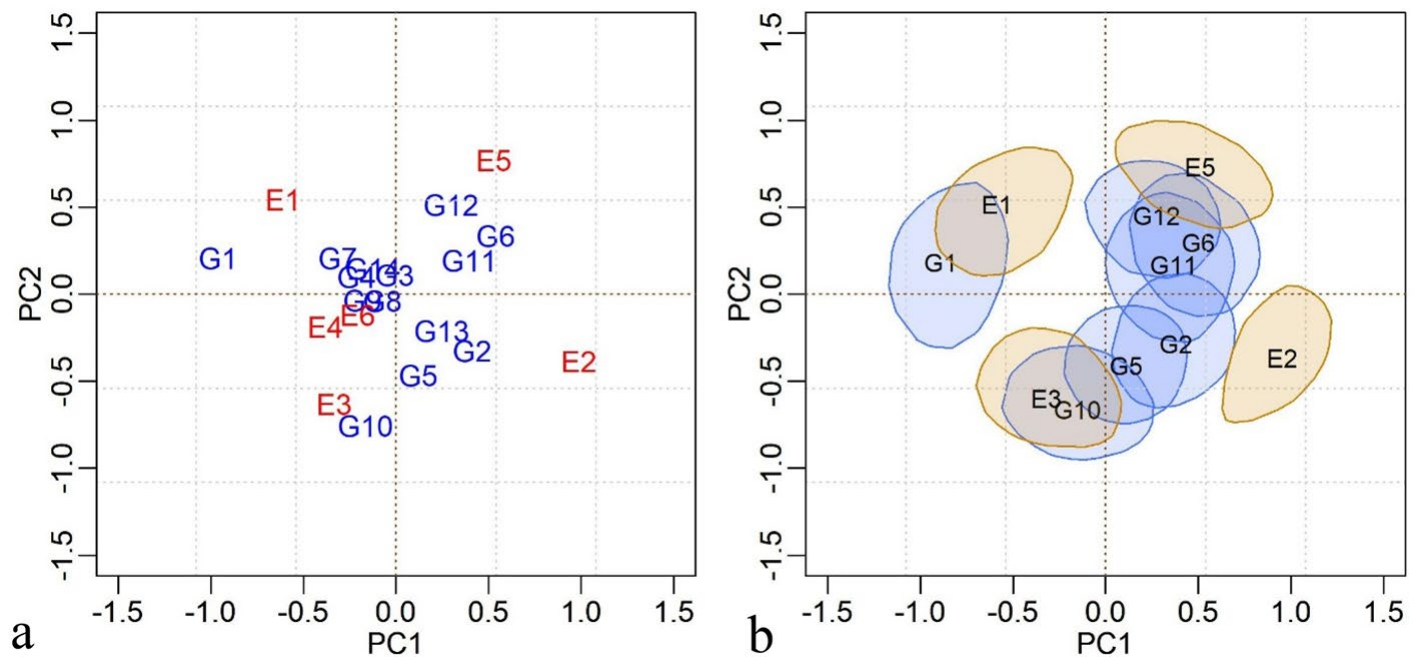
Biplot of the BAMMI model: **a** Average genotypic and environmental scores and **b** Regions of bivariate credibility (95%) for data from competition trials with carioca beans in the Agreste-Sertão of Pernambuco.

The adaptability and stability analysis is performed by biplot interpretation for genotypic and environmental scores. Genotypes and environments whose credibility regions for the scores encompassed the origin are considered stable^11^. Thus, the lineages CNFC 15460 (G3), CNFC 15462 (G4), CNFC 15497 (7), CNFC 15504 (G8), CNFC 15507 (G9), and cultivars BRS Notável (G13) and BRS Pérola (G14) make up a homogeneous subgroup of genotypes that did not significantly contribute to the GxE interaction, indicating wide adaptation (Fig. 5a).

To simplify the interpretations, only the credibility regions that do not encompass the origin (0,0) were represented, and the respective genotypes (or environments) are considered not stable, in other words, they have a significant contribution to interaction (Fig. 5b).

The bivariate regions of credibility for the genotypic and environmental scores, implemented in the biplot, are used to analyze the effect of the GxE interaction, in which overlaps between them are used to interpret the adaptability and stability of the materials.

The visual analysis of the regions in the biplot allows us to suggest adaptability of genotypes to specific locations, as is the case of the cultivar IPR 139 (G1) to the Arcoverde municipality (E1,E4), from the genotypes CNFC 15480 (G6) and CNFC 15534 (G11), to the Belém de São Francisco municipality (E5 and E2), and from the genotype CNFC 15513 (G10) to the São João municipality (E3, E6) (Fig. 5b).

### Predictive evaluation of models

**Figure 6:**
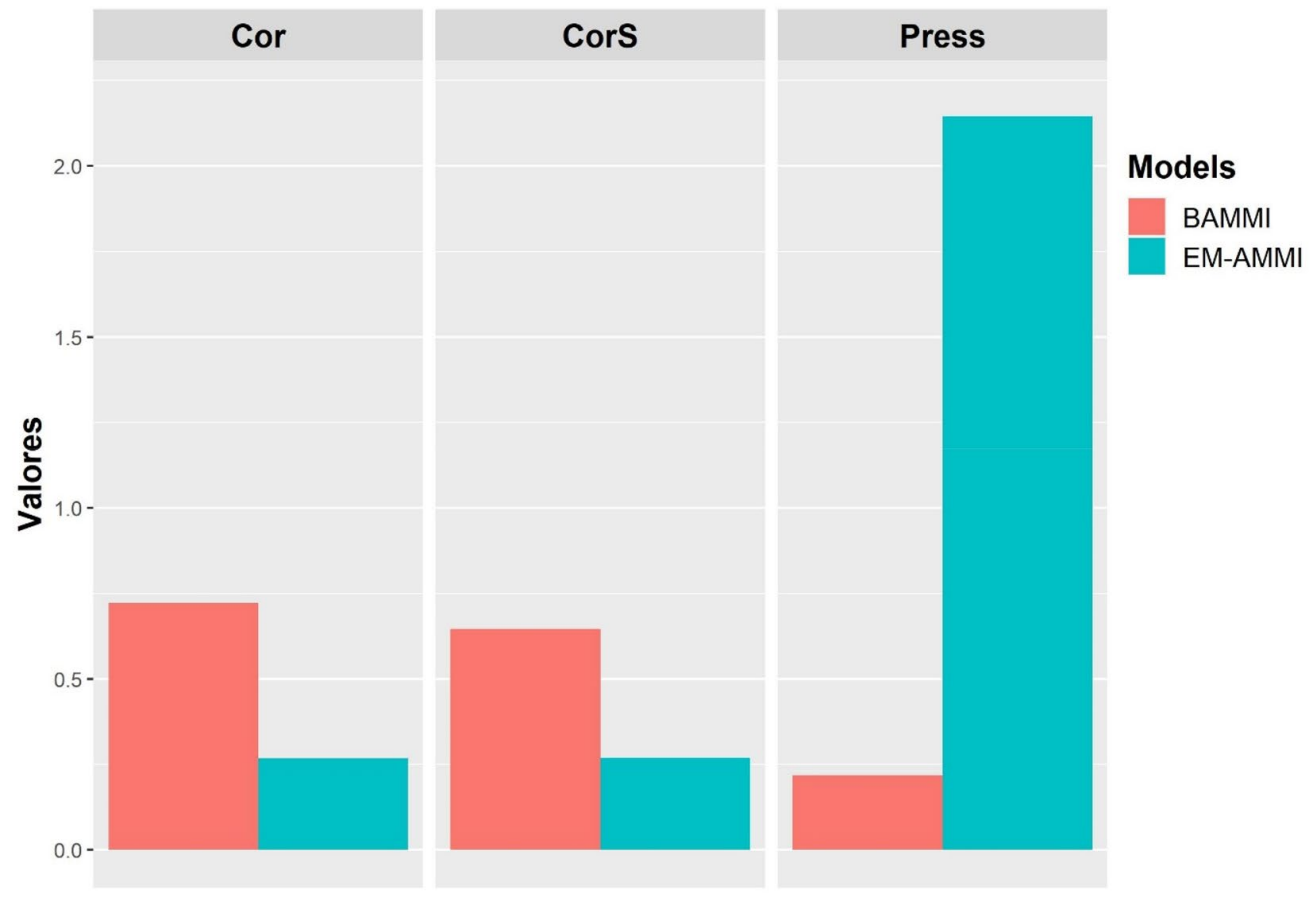
Average Pearson Correlation (Cor), Spearman Correlation (CorS) and PRESS for EM-AMMI and BAMMI models (10% unbalance).

The results of the prediction analysis for the BAMMI and EM-AMMI models are shown in Figs. 5 and 6, for unbalance of 10% and 20%, respectively. The best performance of the model is indicated by the lowest value in relation to the PRESS criterion, and by the highest value in relation to Pearson's correlation (Cor) and Spearman's correlation (CorS)^12^.

**Figure 7:**
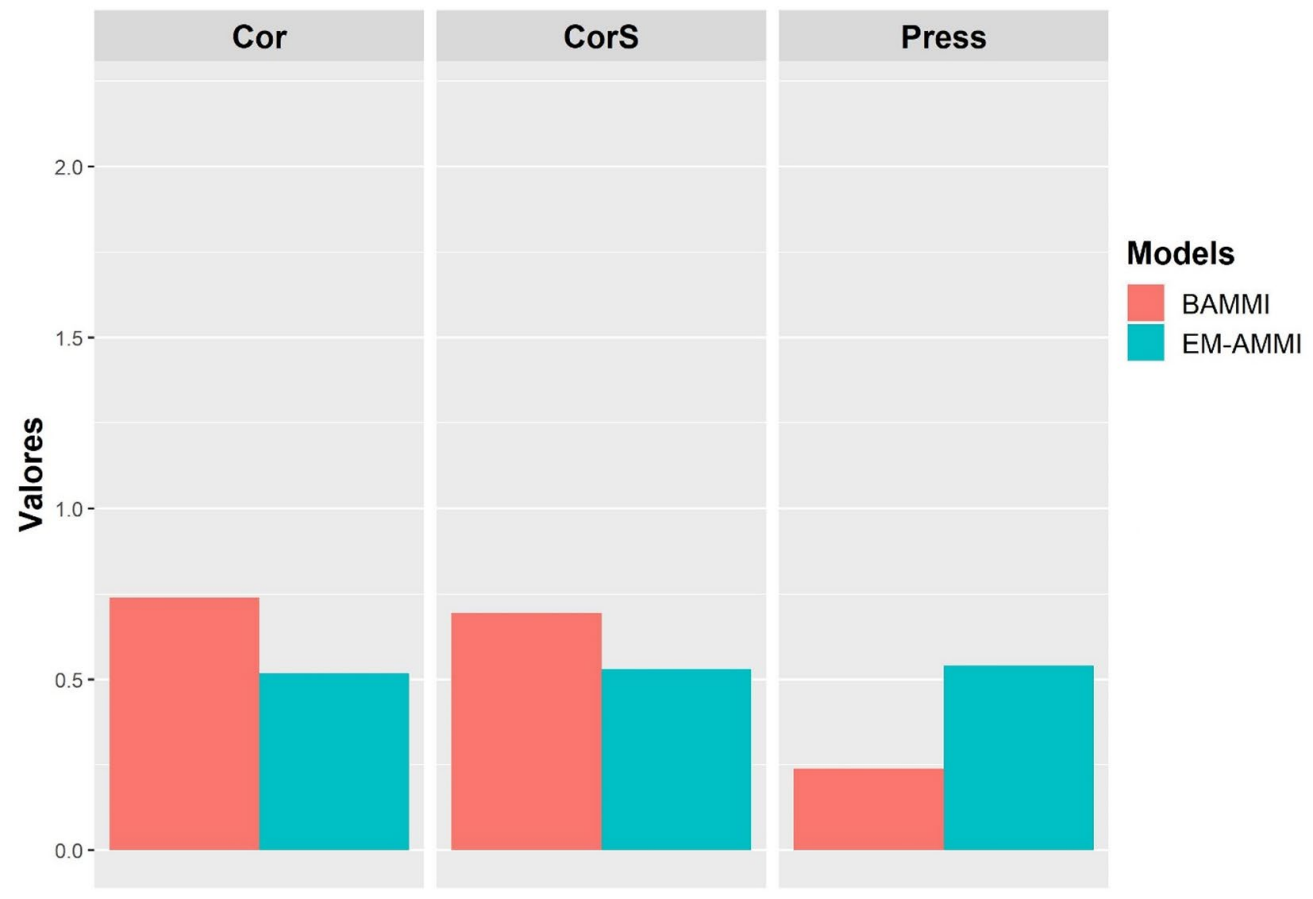
Average Pearson Correlation (Cor), Spearman Correlation (CorS) and PRESS for EM-AMMI and BAMMI models (20% unbalance).

The graphical analyzes demonstrate that the predictive accuracy between the models differed between the imbalance scenarios, however, both showed an advantage for the BAMMI model in all cross-validation criteria (Figs. 6 and 7), being more accentuated in the scenario of lower imbalance (Fig. 6). Similar results were observed by Romão et al.^8^, who identified the advantage of Bayesian models in unbalanced scenarios.

## Discussion

### AMMI-classic analysis

The distinction of genotypes that showed specific interaction with the municipality of Belém de São Francisco it may have occurred due to operational problems during the the management of crops. It is worth noting that the average yields in Belém de São Francisco in both years were the highest among the other environments (Table 7), fact possibly linked to the number of genotypes with specific adaptability to this municipality and to having an irrigation system. A similar consideration can be made in relation to the Arcoverde municipality.

**Table 7:** General averages referring to grain productivity (kg ha^-1^) of carioca bean genotypes, evaluated in the state of Pernambuco, from 2014 to 2015.

	Environments	Harvest	Average		Environments	Harvest	Average
E1	Arcoverde	2014	2265.17	E4	Arcoverde	2015	2237.74
E2	B. S. Francisco	2014	2278.21	E5	B. S. Francisco	2015	3220.24
E3	São João	2014	1453.99	E6	São João	2015	1766.37

The São João municipality (E3 and E6) had the lowest average productivity among the environments (Table 7), however, it is possible to visualize in the biplot that it was the *municipality* that presented the greatest consistency in relation to the effect of the GxE interaction in the years considered (Fig. 2). The lower averages can be explained by the low rainfall (Table 1 and Fig. 1), since the experiments at this location were conducted under rainfed conditions, furthermore, the municipalities have different types of soil and the cultivation in São João depends, essentially, of the amount of organic matter put in each year.

Regarding the Arcoverde municipality (E1 and E4), although consistency has not been maintained over the years, it is possible to observe that the genotype G1 could be indicated and would allow the exploration of the positive effect of the GxE interaction. The Belém de São Francisco municipality (E2 and E5) was the one that showed the greatest inconsistency, in relation to GxE interaction, in the two years evaluated, evidencing the performance of the complex type of interaction. Even so, genotypes G6 and G11 would be those with greater adaptability to this location (Fig. 2).

It is common for edaphoclimatic variations between different years to cause fluctuations in genotypic and environmental scores, masking the graphic patterns identified in the biplot, and therefore it is difficult to observe genotypes that maintain their performance for the same location in different years. Thus, the variation between the interaction effects in different years for a specific location may be like the effects for different locations, due to changes between years^9^.

The recommendation of cultivars with specific adaptation characterizes an efficient way that breeders use to take advantage of the GxE interaction, benefiting environments with conditions normally unfavorable to cultivation, as well as promoting an increase in average productivity in environments with good cultivation conditions, when using materials that could make the most of the interaction effect^40,41^.

The heterogeneity of the municipalities of Agreste-Sertão of Pernambuco directly influences productivity, promoting and justifying the variation in average productivity observed between environments, as observed in the work of Souza et al.^42^,Santos et al.^43^,Santos et al.^44^ and Lima et al.^1^. This heterogeneity, in addition to being confirmed in the analysis of variance (Table 3), it is evident in the description of the edaphoclimatic characteristics of each municipality (Table 1). It is worth noting that the evaluated pre-cultivars showed average productivity above the state (464.12 kg ha^-1^), regional (397.29 kg ha^-1^) and national (990 kg ha^-1^) averages^3^.

### AMMI-bayesian analysis

The predictions of the BAMMI model slightly smaller than the respective minimum squares solutions (Table 6) is due to the shrinkage effect when assuming a common population for genotypes (random effects), in addition to being a common feature of Bayesian methods, corroborating what was observed by Oliveira et al.^11^ and Oliveira et al.^12^.

When considering the effects of genotypes as random, it allows for variance components to be estimated and for kinship coefficients to be incorporated in the AMMI analysis. This leads to more accurate estimates of the genetic values of the genotypes and, consequently, it offers more realistic estimates of the true genotypic value.

The stage of evaluation and selection of pre-cultivars in competition trials requires both the skill of the breeder as well as statistical tools that allow the correct interpretation of phenotypic data. In this sense, the use of shrinkage effect predictors is an excellent choice for plant breeding programs, because they are more accurate and facilitate the classification of genetic materials according to their genotypic values, minimizing the residual effects of environments present in the data^12,19^.

The analysis of the main effect of the genotype is very important for the selection and recommendation of cultivars, but it is essential that the effects of the GxE interaction are jointly considered, to evaluate the adaptability and stability of the materials^19^.

The credibility regions obtained by the BAMMI model promote the elimination of subjective average scores close to the central point of the biplot, leading to greater precision to infer about genotypic and environmental stability^45^. This is evident in the interpretation of the biplots (fixed and Bayesian), in which, in the Bayesian analysis, the environments Arcoverde-2015 (E4) and São João-2015 (E6) do not significantly contribute to the interaction (Fig. 5b).

Among the genotypes that stand out in terms of the main effect only BRS Notável can be considered stable and, therefore, has a wide recommendation (Fig. 5b). This interpretation is not direct with respect to the biplot of the frequentist model and would be a risky assumption in the context of classical analysis. However, using the 95% credibility level of the Baysean model, it is possible to safely identify G13 as stable, that is, this genotype has no important contribution to the GxE interaction.

The cultivar BRS Notável was used as a witness in the experiments because it has a recommendation for cultivation in the state of Pernambuco, in addition to other Brazilian states, as well as the other three cultivars mentioned in this work. However, BRS Notável was the only one that combined positive genotypic effect and stability, reinforcing its viability and potential for the Agreste-Sertão region of Pernambuco.

Credible regions for genotypic and environmental scores ensure that discrimination of genotypes and environments is performed with greater precision, grouping genotypes and environments into homogeneous subgroups that show the same patterns of interaction. The selection of genotypes with an associated level of credibility helps plant breeders in decision making, leading to the recommendation of more consistent cultivars, and reducing the costs of breeding programs^9,11^.

Generally, genotypes that have less than zero main effects been not recommended, given that they have a response below the population average^9^. However, in relation to the pre-cultivars evaluated in this work, it can be seen that all had an average productivity significantly higher than the regional average (397.29 kg ha^-1^), as shown in Table 8, it is therefore pertinent to indicate these materials for the places that had good interaction, according to the adaptability analysis through the credibility regions.

**Table 8:** Carioca bean genotypes evaluated in competition trials in the years 2014 and 2015 and average grain productivity (kg ha^-1^).

IG	Genotype	Prod	IG	Genotype	Prod
G1	IPR 139	2224.03	G8	CNFC 15504	2117.92
G2	CNFC 15458	2350	G9	CNFC 15507	1907.08
G3	CNFC 15460	2260.55	G10	CNFC 15513	1875.69
G4	CNFC 15462	2219.58	G11	CNFC 15534	2125
G5	CNFC 15475	2029.30	G12	BRS Estilo	2632.36
G6	CNFC 15480	2586.25	G13	BRS Notável	2453.61
G7	CNFC 15497	1818.47	G14	BRS Pérola	2250.83

Overlaps between regions indicate homogeneous subgroups in terms of the interaction effect, demonstrating that the genotypes present similar behavior in the studied environments. Thus, bivariate regions are an excellent tool for recommending genotypes to specific environments^7^.

The lineage CNFC 15480 (G6) deserves to be highlighted because it presented a positive genotypic effect (Table 6) and productivity slightly higher than that of the witnesses (Table 8), except for BRS Estilo, corroborating its indication for the municipality of Belém de São Francisco, as it demonstrated specific adaptability for that location (Fig. 5b).

The configurations of the midpoints of genotypic and environmental scores in the biplots of the AMMI frequentist and AMMI Bayesian models were similar. However, the analysis of the credibility regions by the biplot of the BAMMI model provided a clearer organization of the specific interactions of the genotypes with certain environments, allowing you to relate materials more firmly to specific locations, despite the differences perceived in the different years, which were probably caused by the edaphoclimatic variations of the municipality. Furthermore, the incorporation of inference to the biplot allowed us to identify environments and genotypes that do not have important contributions to the GxE interaction.

The Agreste-Sertão of Pernambuco presents great climatic variations between harvests; thus, the same locality can vary a lot in different years. However, the BAMMI method showed efficiency in grouping genotypes with specific adaptation to certain locations, even in different years, corroborating the results obtained by Bernardo Júnior et al.^9^ who verified the good ability of this method to discriminate the environments within the different seasons.

## Predictive evaluation of models

In general, the EM-AMMI model showed good performance in slightly accentuated imbalance scenarios, such as those used in this work, showing good ability to predict the GxE interaction, without requiring sophisticated statistical methods, high computational demand, or estimation of complex parameters. According to Paderewski and Rodrigues^36^, the EM algorithm is great for imputing missing data, since its iterative process is done in the GxE matrix, converging the imputation of the interaction in a few steps, contributing to the quality of the fit, which could justify the good performance of the EM-AMMI models in scenarios with losses of up to 33%.

Both models showed that they are robust to data loss in the analyses, however, the BAMMI model was superior in predictive accuracy and shows great potential of the technique in the study of GxE interaction and in the prediction of missing genotypes. The need to deal with unbalanced data is a recurring inconvenience in adaptability and stability studies because environmental variations and other unpredictable factors in field experiments often lead to plot loss. BAMMI analysis naturally handles unbalanced datasets and still allows dealing with uncertainty in biplots, being highly indicated for genotype evaluation^9^.

Compared to conventional methods, the BAMMI methodology has the disadvantage of being more time consuming and requiring greater demand for computational resources, but the greater precision in the inference process, added to the other advantages mentioned throughout this work, offered by this method, justify its use. The Bayesian analysis of multiplicative models has gained more space in the analysis of multi-environmental data, due to its benefits and because of the computational progress that has mitigated the difficulty of performing the analyzes^10^.

Reinforcing the advances that facilitate Bayesian analysis, emphasizing the BAMMI model, it is worth noting that the ammiBayes package, recently developed by Oliveira et al.^23^ and pioneered in this work, allows the user of the statistical program to perform the entire inference process in a reasonably simple and practical way.

## Conclusions

The cultivar BRS Notável (G13) is able to capitalize on positive interactions with the environments, being classified as stable and recommended with wide adaptation for the Agreste-Sertão of Pernambuco; Arcoverde and São João are the municipalities that present greater stability for the effects of the GxE interaction, evidenced by the BAMMI analysis through the credibility regions.

The recommendation of cultivar IPR 139 (G1) allows exploring the positive effect of GxE in Arcoverde, while in Belém de São Francisco the pre-cultivars CNFC 15480 (G6) and CNFC 15534 (G11) are used; in São João, the genotype CNFC 15513 (G10) shows greater adaptability.

The BAMMI analysis is more capable of identifying environments and genotypes that do not have important contributions to the GxE interaction; this model exhibits superior predictive ability compared to the EM-AMMI model, for unbalance scenarios up to 10% and 20%.

## Data Availability

All data generated or analyzed during this study are included in this published article.
